# Influencing factors of acute kidney injury in elderly patients with diabetic nephropathy and establishment of nomogram model

**DOI:** 10.3389/fendo.2024.1431873

**Published:** 2025-01-30

**Authors:** Ganlin Wu, Yanli Ye, Meirong Xu, Yanxia Zhang, Zuopeng Lu, Lv Huang

**Affiliations:** ^1^ School of Clinical Medicine, Xianning Medical College, Hubei University of Science and Technology, Xianning, China; ^2^ National Demonstration Center for Experimental (General Practice) Education, Xianning Medical College, Hubei University of Science and Technology, Xianning, China; ^3^ Department of Internal Medicine, The Second Affiliated Hospital of Hubei University of Science and Technology, Xianning, China; ^4^ Department of Geriatrics, The Second Affiliated Hospital of Hubei University of Science and Technology, Xianning, China; ^5^ Department of Nephrology, Tongren Hospital of Wuhan University (Wuhan Third Hospital), Wuhan University, Wuhan, Hubei, China; ^6^ Department of Surgery, People’s Hospital of Tongcheng County, Xianning, China; ^7^ College of Pharmacy, Hubei University of Science and Technology, Xianning, China

**Keywords:** acute kidney Injury, elderly diabetic nephropathy, influencing factors, nomogram, model

## Abstract

**Purpose:**

To explore the influencing factors of acute kidney injury in elderly patients with diabetic nephropathy and to construct a nomogram model.

**Methods:**

The research subjects were 680 patients with type 2 diabetic nephropathy admitted to our hospital. The patients were included from May 2018 to August 2023. Patients with acute kidney injury were used as the merge group (n=50), and patients without unmerge group (n=630) was included. The prevalence and predisposing factors of acute kidney injury in diabetic nephropathy were analyzed, multivariate logistic regression were used to analyze the influencing factors of acute kidney injury in patients, and a nomogram risk prediction model was established based on risk factors for verification.

**Results:**

Analysis of the factors of acute kidney injury in diabetic nephropathy found that severe infection was the main trigger, accounting for 40.00%, followed by nephrotoxic antibiotics and severe heart failure. The age, urine microalbumin-to-creatinine ratio (ACR), blood urea nitrogen (BUN), uric acid(UA), and cystatin C (CysC) levels of patients in the combined acute kidney injury group were significantly higher than those in the unmerge group (*P*<0.05), and the left ventricular ejection fraction (LVEF) and epidermal growth factor receptor (eGFR) levels were significantly lower than those in the unmerge group (*P*<0.05). Age, ACR, and CysC levels are independent risk factors for acute kidney injury in diabetic nephropathy, and LVEF and eGFR are independent protective factors (*P*<0.05). The C-index of the nomogram risk prediction model in predicting acute kidney injury in diabetic nephropathy is 0.768 (95% CI: 0.663-0.806), and the calibration curve tends to the ideal curve; the prediction threshold is >0.18, and the nomogram risk prediction model provides a clinical net benefits, and clinical net benefits were higher than independent predictors.

**Conclusion:**

The establishment of a nomogram model for acute kidney injury in elderly patients with diabetic nephropathy based on age, ACR, CysC, LVEF, and eGFR has a good predictive effect, which can help doctors more accurately assess the patient’s condition and provide a basis for formulating personalized treatment plans.

## 1 Introduction

Diabetes is one of the chronic diseases that seriously threatens human health around the world. The kidney is one of the most commonly affected organs in diabetes. Diabetic nephropathy is a common microvascular complication of diabetes. The onset is insidious. In the early stages of kidney damage, there may be no symptoms. As the disease progresses, foamy urine, high blood pressure, and edema (swelling of feet, ankles, hands, or eyes) may gradually occur (1, 2). If it develops into end-stage renal disease, which is kidney failure, water, electrolyte, acid-base balance disorders and anemia will occur. The pathogenesis of diabetic nephropathy is complex and involves multiple factors such as hyperglycemia, hypertension, lipid metabolism disorders, and inflammatory reactions. With the increasing number of diabetic patients, diabetic nephropathy plays a vital role in promoting the occurrence of end-stage renal disease (3, 4). In our country, the prevalence of diabetic nephropathy is high, which brings a heavy burden to society and families (5). Acute kidney injury refers to a sudden decline in kidney function within a short period of time (usually 72 hours), leading to clinical manifestations such as azotemia, water and electrolyte disorders, and acid-base balance disorders. Patients with diabetic nephropathy are prone to acute kidney injury and have a poor prognosis (6). Acute kidney injury aggravates the condition of diabetic nephropathy, and patients need treatment such as dialysis or kidney transplantation, increasing the patient’s risk of death. Therefore, studying the influencing factors of acute kidney injury in elderly patients with diabetic nephropathy will help us better understand the characteristics of such patients and provide scientific basis for clinical prevention and treatment (7, 8). At the same time, by establishing a prediction model, patients can be warned of the risk of acute kidney injury in advance, providing a reference for clinicians to formulate individualized treatment plans. The nomogram model is a prediction model based on logistic regression analysis, which can predict the risk of acute kidney injury in diabetic nephropathy by integrating the relative importance of multiple influencing factors. Some studies have found (9, 10) that the nomogram model has certain accuracy in predicting acute kidney injury in diabetic nephropathy and can be used to guide clinicians in formulating preventive strategies and treatment plans. This study aims to explore the factors that influence the occurrence of acute kidney injury in elderly patients with diabetic nephropathy, which will help improve the prevention and treatment effect and reduce the mortality rate of patients.

## 2 Materials and methods

### 2.1 General information

**Figure 1:**
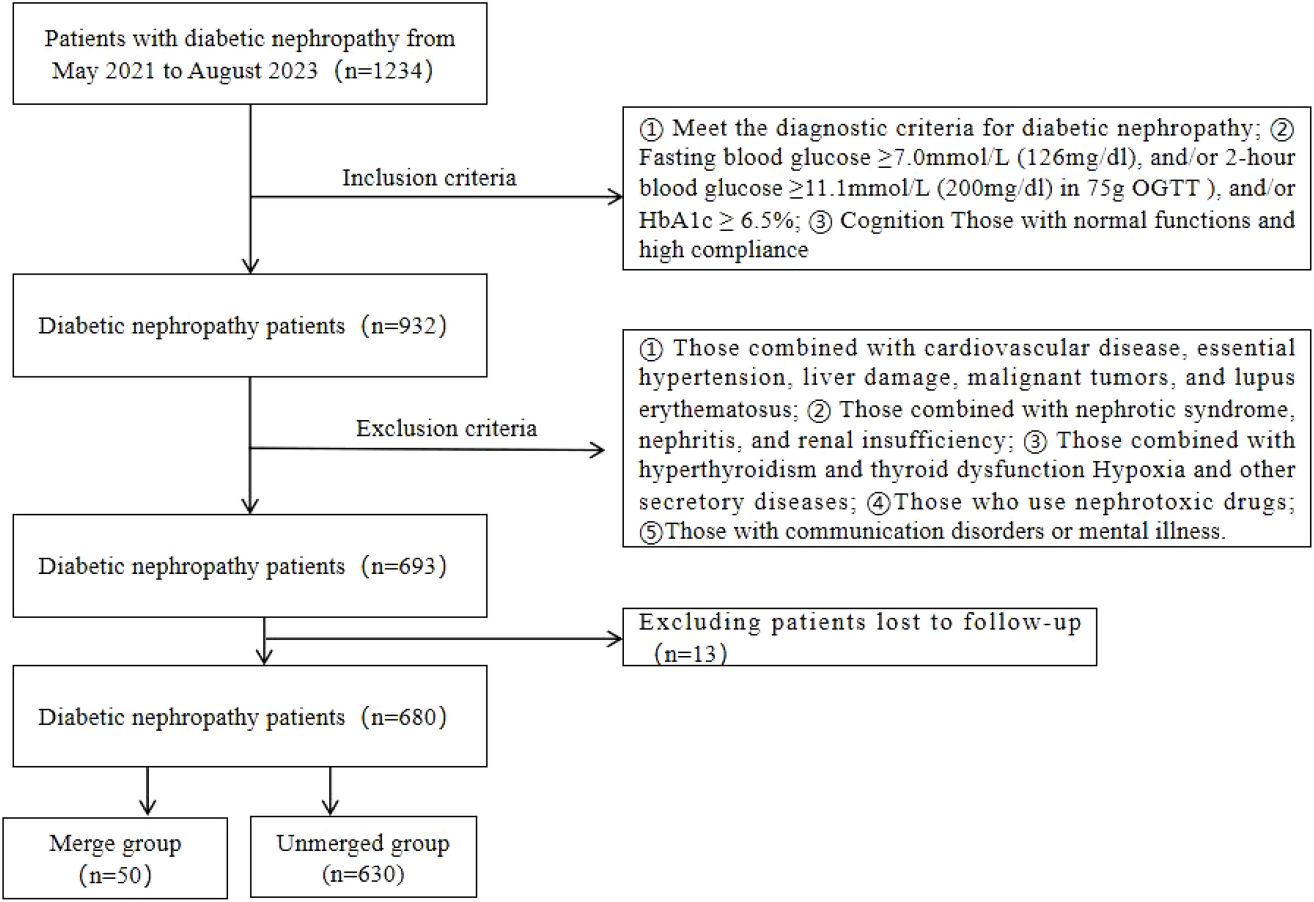
Literature screening flow chart.

The research subjects were 680 patients with type 2 diabetic nephropathy admitted to our hospital. The patients were included from May 2018 to August 2023. Patients with acute kidney injury were included in the merged group (n =50), unmerged group (n=630). Inclusion criteria: ① Diagnostic criteria for diabetic nephropathy are based on the chronic kidney disease assessment and clinical practice guidelines issued by KDIGO (Kidney Disease Improving Global Outcomes) in 2012. Urine microalbuminuria should be tested three times within three months, with at least two urine microalbuminurias ≥ 30 mg/24 h (11); ② Fasting blood glucose ≥7.0mmol/L (126mg/dl), and/or 2-hour blood glucose ≥11.1mmol/L (200mg/dl) in 75g OGTT), and/or HbA1c ≥ 6.5%; ③ Cognition Those with normal functions and high compliance; exclusion criteria: ① Those combined with cardiovascular disease, essential hypertension, liver damage, malignant tumors, and lupus erythematosus; ② Those combined with nephrotic syndrome, nephritis, and renal insufficiency; ③ Those combined with hyperthyroidism and thyroid dysfunction Hypoxia and other secretory diseases; ④Those who use nephrotoxic drugs; ⑤Those with communication disorders or mental illness. This study was approved by the hospital ethics committee. Signed informed consent were also obtained from all participants. According to the inclusion and exclusion criteria, 693 patients with diabetic nephropathy were screened. After excluding 13 patients who were lost to follow-up, 680 patients with diabetic nephropathy were finally obtained.The screening process is shown in **Figure 1**.

### 2.2 Diagnostic criteria for acute kidney injury

Refer to the AKI diagnostic criteria proposed by KDIGO: serum creatinine increases by more than 1.5 times the baseline, and occurs within 7 days of the clear or inferred increase in serum creatinine level. The reference range of serum creatinine is 53-106μmol/L for adult males and 44-97μmol/L for adult females.

### 2.3 Data collection

General information: Age, gender, systolic and diastolic blood pressure were collected for all patients.

Laboratory indicators: eGFR, glycated hemoglobin (HbAlc), serum albumin, ACR, LVEF, BUN, serum creatinine (Scr), UA, and CysC levels.

Nomogram analysis: Establish a nomogram model: Taking all patients as the research subjects, based on the results of logistic regression analysis, factors that have a significant impact on acute kidney injury in elderly patients with diabetic nephropathy are screened out and included in the nomogram model. The best model was determined through receiver operating characteristic curve (ROC) analysis.

Validate the nomogram model: Apply the established nomogram model to predict the risk of acute kidney injury. The predictive effectiveness of the model was evaluated by comparing the number of patients who actually developed acute kidney injury with the number of patients who were predicted to develop acute kidney injury. Draw a calibration chart for verification and evaluate the accuracy of the risk model.

### 2.4 Methods of collecting and collating data

By reviewing the patients’ medical records, the collected data were collated and statistically analysed using Excel sheets. For missing values or outliers, we used the mean to fill in or exclude to ensure the accuracy and reliability of the data. By analysing the collated data, we aimed to explore the factors affecting the occurrence of acute kidney injury in elderly patients with diabetic nephropathy, and to provide a reference basis for clinical prevention and treatment.

### 2.5 Statistical methods

The enumeration data in this group of studies are expressed as [cases (%)], and the *x* ^2^ test is used. The measurement data are all in the form of ( $\bar{x}$ ± *s)* form, an independent sample *t* test was used between the two groups. The influencing factors of acute kidney injury in elderly patients with diabetic nephropathy were analyzed using multi-factor logistic regression, and a nomogram risk prediction model was established based on the risk factors. Bootstrap self-sampling method was used for internal testing. The predictive ability of the model was tested through the receiver operating characteristic curve (ROC curve), C-index and calibration curve are used for verification. SPSS 23.0 software was used for statistical data analysis, and *P* < 0.05 was considered as a statistically significant difference.

## 3 Results

### 3.1 Analysis of the prevalence of acute kidney injury in diabetic nephropathy

A total of 50 patients with acute kidney injury occurred among 680 patients with diabetic nephropathy, with an incidence rate of 7.35%; there were differences in the prevalence of acute kidney injury among patients with diabetic nephropathy of different ages and eGFR levels (*x* ^2^ = 7.722, 18.987, *P <*0.05). See **Table 1**.

**Table 1:** Prevalence analysis of acute kidney injury in diabetic nephropathy.

Normal information	Number of examples	Prevalence of acute kidney injury	*P*
gender			0.614
male	468 (68.82)	36 (72.00)	
female	212 (31.18)	14 (28.00)	
age			0.005
<60	246 (36.18)	9 (18.00)	
≥60	434 (63.82)	41 (82.00)	
eGFR [mL (min·1.73m ^2)^]			<0.001
<30	135 (19.85)	20 (40.00)	
30~59	221 (32.50)	19 (38.00)	
≥60	324 (47.65)	11 (22.00)	

### 3.2 Analysis of influencing factors of acute kidney injury in diabetic nephropathy

Analysis of the influencing factors of acute kidney injury in diabetic nephropathy found that severe infection was the main trigger, accounting for 40.00%, followed by nephrotoxic antibiotics and severe heart failure. The details are shown in **Table 2**.

**Table 2:** Analysis of influencing factors of acute kidney injury in diabetic nephropathy.

Predisposing factors	Number of examples	Proportion
severe infection	20	40.00
nephrotoxic antibiotics	7	14.00
severe heart failure	6	12.00
contrast agent	5	10.00
acute cerebrovascular accident	4	8.00
proton pump inhibitor	3	6.00
acute cerebrovascular accident	5	10.00
total	50	100.00

### 3.3 Analysis of clinical data of the two groups

CysC levels of patients in the combined acute kidney injury group were significantly higher than those in the unmerge group (*P <*0.05), and the LVEF and eGFR levels were significantly lower than those in the unmerge group (*P <*0.05), see **Table 3**.

**Table 3:** Analysis of clinical data of the two groups.

Factor	Merge group (n=50)	Unmerged group (n=630)	t/χ2	*P*
age	54.56 ± 15.88	44.25 ± 12.85	5.360	<0.001
gender				
male	29	439	2.947	0.086
female	21	191		
The duration of diabetes(Years)	9.10 ± 1.86	9.38 ± 1.65	1.144	0.253
Use ACEI	19 (38.00)	201 (31.90)	0.786	0.375
Use ARB	4 (8.00)	53 (8.41)	0.010	0.919
Use statins	23 (46.00)	247 (39.21)	0.893	0.345
Systolic blood pressure (mmHg)	140.25 ± 22.15	141.36 ± 26.96	0.284	0.777
Diastolic blood pressure (mmHg)	82.25 ± 8.24	80.44 ± 9.38	1.324	0.186
HbAlc (g/L)	7.88 ± 1.29	7.78 ± 2.01	0.346	0.729
Serum albumin (g/L)	33.42 ± 8.56	35.15 ± 9.18	1.289	0.198
ACR (g/L)	1924.31 ± 375.30	1238.32 ± 205.71	20.999	<0.001
LVEF(%)	48.03 ± 15.28	56.98 ± 19.77	3.127	0.002
BUN (mmol/L)	11.52 ± 4.16	9.44 ± 3.25	3.452	0.007
Scr ( mmol/L)	105.42 ± 12.33	107.88 ± 15.31	1.108	0.268
UA(mmol/L)	426.36 ± 54.65	361.25 ± 28.44	8.331	<0.001
CysC (mg/L)	2.88 ± 0.79	2.41 ± 0.52	4.135	<0.001
eGFR [mL(min·1.73m ^2)^]	33.42 ± 18.29	51.25 ± 24.26	5.082	<0.001

### 3.4 Multi-factor logistic regression analysis

Taking the statistically significant indicators in **Table 3** as independent variables and whether acute kidney injury occurs in diabetic nephropathy as the dependent variable, the results show that age, ACR, and CysC levels are independent risk factors for acute kidney injury in diabetic nephropathy, and LVEF and eGFR are independent protective factor (*P <*0.05). See **Supplementary Table S1**.

### 3.5 Construct a nomogram risk prediction model

**Figure 2:**
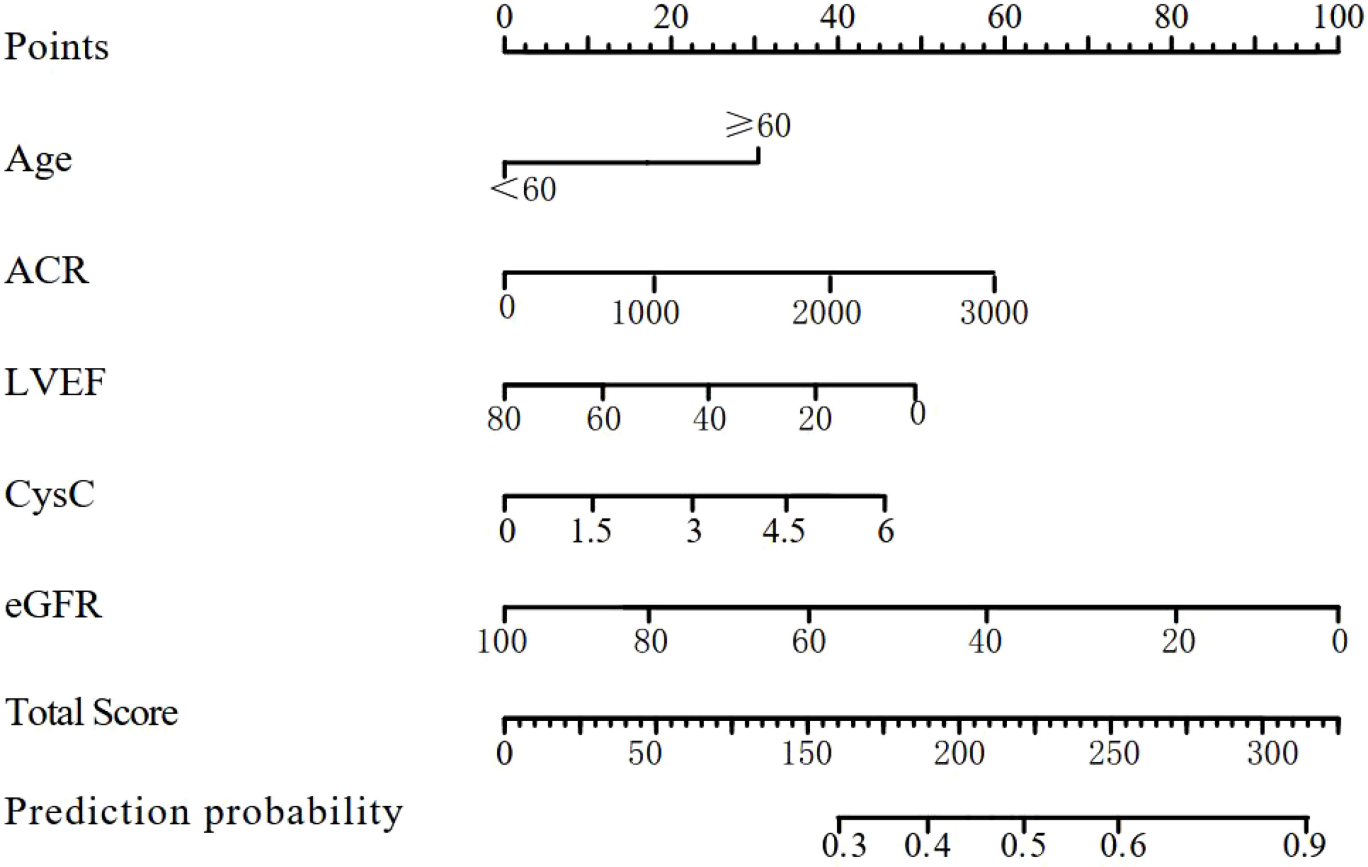
Nomogram risk prediction model for acute kidney injury in diabetic nephropathy.

After the multicollinearity test, the VIF range of the three variables RRI, MFI, and PVD was 1.316-2.501, indicating that there was no multicollinearity problem between them. Age, ACR, CysC, LVEF, and eGFR were used as factors to construct the nomogram risk prediction model. The nomogram risk prediction situation is shown in **Figure 2**.

### 3.6 Calibration curve and decision curve

**Figure 3:**
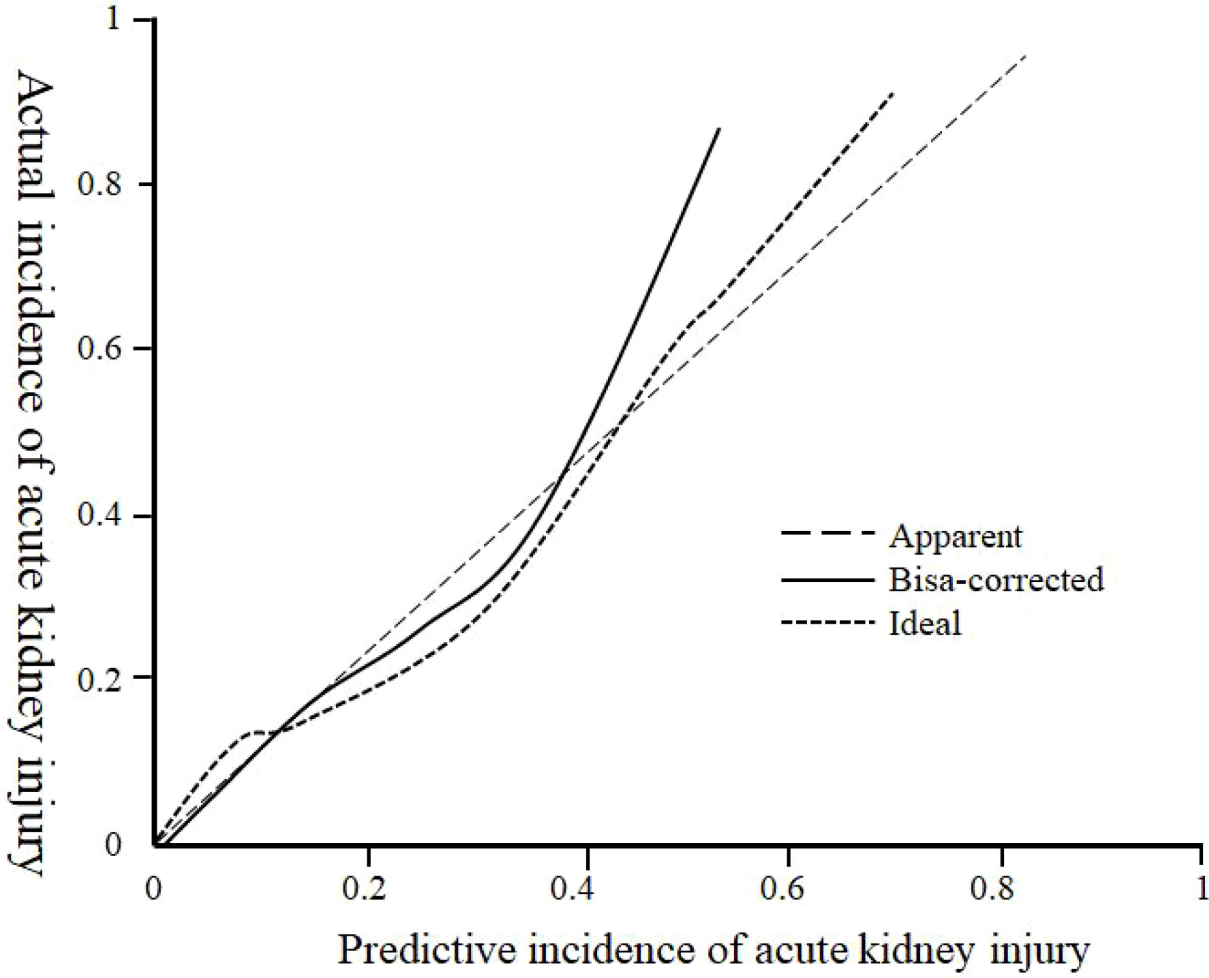
Calibration curve of the nomogram risk prediction model.

**Figure 4:**
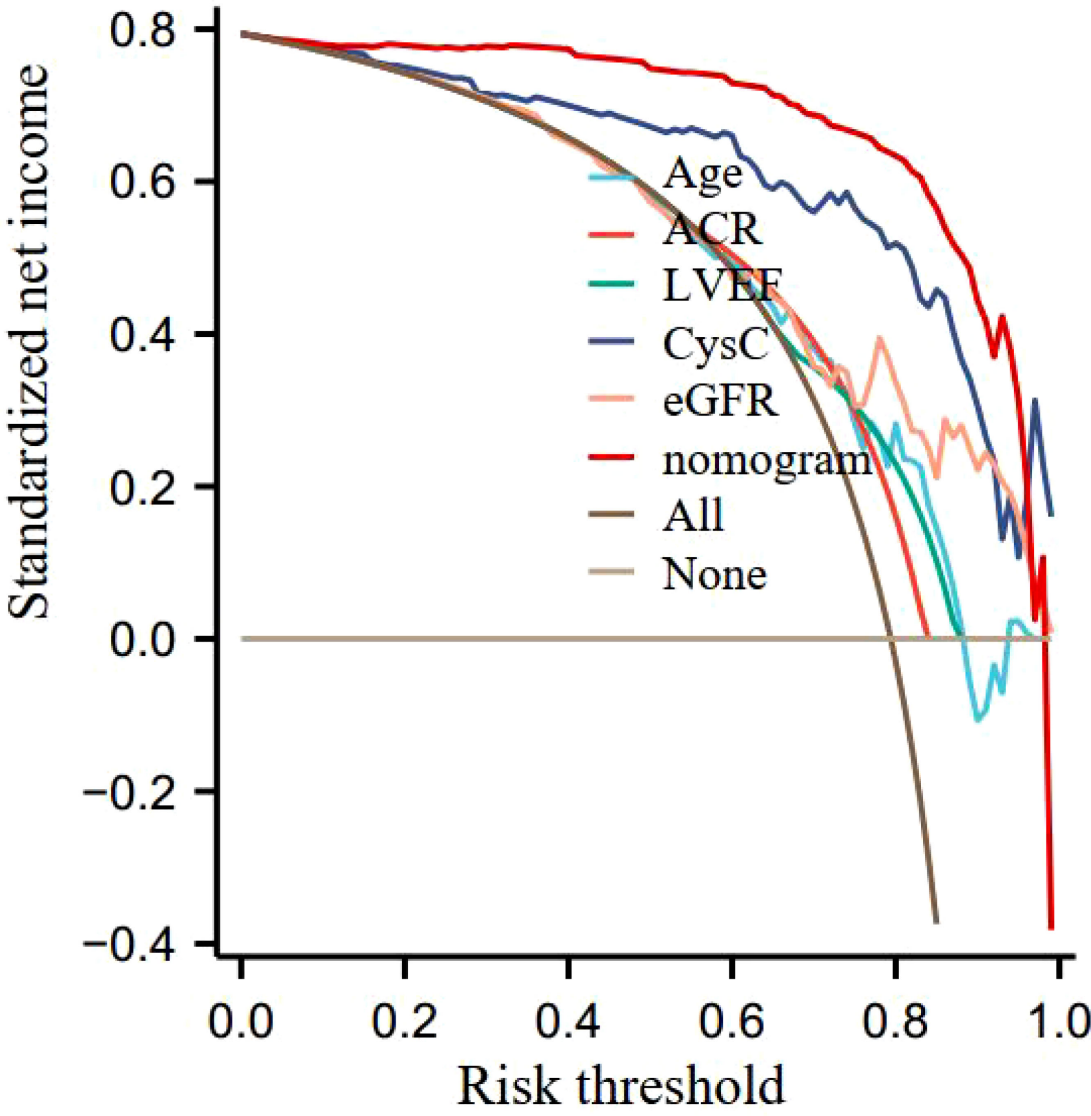
Nomogram risk prediction model decision curve.

the nomogram risk prediction model for predicting acute kidney injury in diabetic nephropathy is 0.768 (95% CI: 0.663-0.806), and the calibration curve tends to the ideal curve, see **Figure 3**; the prediction threshold is >0.18, and the nomogram risk prediction The model provides clinical net benefits, and the clinical net benefits are all higher than independent predictors, see **Figure 4**.

## 4 Discussion

Diabetic nephropathy is a complication of diabetes, which mainly manifests as decreased renal filtration function, leading to symptoms such as proteinuria and edema. As diabetes progresses, renal lesions gradually worsen and renal function gradually decreases, which may eventually lead to renal failure (12–14). Acute kidney injury is one of the important factors of exacerbation of diabetic nephropathy, seriously affecting patients’ quality of life and prognosis. Therefore, studying the influencing factors of acute kidney injury in elderly patients with diabetic nephropathy is of great significance for the prevention and treatment of diabetic nephropathy. Understanding the influencing factors of acute kidney injury in elderly patients with diabetic nephropathy will help us develop more effective prevention and treatment strategies and reduce the risk of mortality and reduced quality of life in patients.

In this study showed that age, ACR, and CysC levels are independent risk factors for acute kidney injury in diabetic nephropathy, and LVEF and eGFR are independent protective factors. The aging population of society is growing rapidly, and the elderly are also at high risk of chronic kidney disease. Due to reduced renal reserve function, various chronic diseases and comorbidities in the elderly, the elderly, especially elderly patients with chronic kidney disease, are highly susceptible to acute kidney injury, and the incidence of acute kidney injury is significantly higher than that of the general population (15–17). The risk of acute renal failure progressing to uremia or death in the elderly is also much higher than that in young adults with kidney disease. As the elderly age, the structure and function of the kidneys change, and aging changes such as glomerulosclerosis, nephron reduction, renal blood vessel wall thickening, renal blood flow and glomerular filtration rate reduction, and renal concentrating function decline gradually occur. As a result, the kidneys of the elderly are prone to acute kidney injury under emergency or injury conditions (18–20). As a clinical test indicator that has been around for many years, ACR is listed as a screening indicator for early kidney disease and a diagnostic and staging standard for chronic kidney disease. It can accurately reflect the status of renal function and improve the clinical value of test results (21). It is helpful to reduce the incidence of complications of diabetic nephropathy and reduce the risk of related diseases. Achieve early diagnosis and early treatment of diabetic nephropathy. Under normal circumstances, the concentration of CysC in serum and plasma is low. When renal function is impaired, the concentration of CysC in the blood changes with the glomerular filtration rate. In renal failure, the glomerular filtration rate decreases, and the concentration of CysC in the blood can increase more than 10 times; if the glomerular filtration rate is normal but the renal tubular function is abnormal, it will hinder the absorption of CysC in the renal tubules. And it is rapidly decomposed, increasing the concentration in urine by more than 100 times (22, 23). In order to prevent diabetes from developing into renal failure, reliable GFR must be used to evaluate the renal function status of diabetic patients. Clinically, SCr is used to evaluate GFR, which lacks sufficient sensitivity for mild renal damage, while CysC can detect mild renal damage. The damage response is sensitive, and regular detection of CysC in diabetic patients can dynamically observe the development of the disease (24, 25). Patients with diabetic nephropathy are also prone to develop cardiovascular disease. Once the ejection fraction of the heart is reduced, it will lead to relative insufficient renal perfusion. Severe infection or severe respiratory failure will further aggravate renal ischemia, leading to a rapid decline in eGFR (26, 27). In addition, drug clearance is reduced after eGFR. Once drugs that may damage the kidneys are used, such as nephrotoxic antibiotics, contrast agents, proton pump inhibitors, etc., the kidney damage will be further aggravated (28–30). Chen et al. (31) found that LVEF is a protective factor for AKI during ECMO support. For every 1% increase in LVEF, the incidence of AKI decreases by 20%. A lower LVEF value before ECMO usually indicates more severe heart failure and shock before starting ECMO support therapy. Decreased cardiac output and hypotension usually mean poor microcirculatory perfusion status. Systemic inflammation is an important mechanism driving AKI and can affect the disease progression and prognosis of patients. Systemic inflammation in elderly patients can aggravate mechanical disorders, microthrombosis, affect cell apoptosis and mitochondrial damage, and induce renal injury. In the context of diabetic nephropathy, the NF-κB signaling pathway is activated, increasing the release of downstream pro-inflammatory factors such as TNF-α, IL-6, IL-1, and IL-18, recruiting monocytes, macrophages, and lymphocytes into the renal tissue, triggering an inflammatory cascade reaction and aggravating renal damage in diabetic nephropathy.

This study established a nomogram model to predict the risk of acute kidney injury in diabetic nephropathy. The C-index of the nomogram risk prediction model in predicting acute kidney injury in diabetic nephropathy is 0.768 (95%CI: 0.663-0.806). The calibration curve tending to the ideal curve; the prediction threshold is >0.18, the nomogram risk prediction model provides clinical net benefits, and the clinical net benefits are higher than independent predictors. This model has the advantage of being easy to understand and operate, and can make individualized predictions based on the patient’s specific situation. Zou et al. (32) found that CysC and eGFR can be used as risk factors for end-stage renal disease in patients with diabetic nephropathy, and a machine learning algorithm based on sAlb, CysC, Hb, eGFR and UTP can effectively predict the incidence of end-stage renal disease.

However, the nomogram model also has certain limitations. First, the establishment of the model relies on existing research data and originates from a single medical institution. There may be differences in regions or specific populations, which makes it impossible to cover all influencing factors, resulting in limited accuracy of prediction results and affecting the external practicality of the nomogram model. Secondly, the nomogram model needs to be updated regularly to reflect the changing research progress and clinical practice. The model prediction is only for reference, and specific clinical decisions still need to be combined with the actual situation of the patient and the professional judgment of the doctor. Finally, the sample size of this study was small, which may have the risk of false negative results. Use with caution before external validation. In future studies, we will increase the sample size to improve the detection ability of different risk factors for AKI and further perform external validation of the nomogram in different populations to further confirm the practicality of this model in a wider range of clinical practice.

In summary, the influencing factors of acute kidney injury in elderly patients with diabetic nephropathy mainly include the patient’s age, ACR, CysC, LVEF, and eGFR. These factors play an important role in the occurrence and development of diabetic nephropathy and provide clinicians with information. Targeted intervention directions. By establishing a nomogram model, the risk of acute kidney injury in diabetic nephropathy was predicted. The model results show that the nomogram model has good prediction effect and has certain clinical application value for early identification of the risk of acute kidney injury in patients with diabetic nephropathy. At the same time, the nomogram model can help doctors more accurately assess the patient’s condition and provide a basis for formulating personalized treatment plans.

## Data Availability

The original contributions presented in the study are included in the article/**Supplementary Material**. Further inquiries can be directed to the corresponding authors.

## Supplementary material

Supplementary Table 1 Multi-factor logistic regression analysis.
