# Supplementary material for: Influencing factors of acute kidney injury in elderly patients with diabetic nephropathy and establishment of nomogram model
Source: Front Endocrinol (Lausanne). 2025 Jan 30;15:1431873. doi: 10.3389/fendo.2024.1431873 (PMC11821420; doi:10.3389/fendo.2024.1431873)
Supplement: Supplementary Table 1 — Multi-factor logistic regression analysis. [file Table1.pdf]

Table S1 Multi-factor logistic regression analysis

index	β	*SE*	*Wald* χ^2 value	*P* value	*OR* value	*95% CI*
age	0.685	0.251	6.453	0.028	2.461	1.854-6.038
ACR	1.458	0.383	14.036	<0.001	4.297	2.028-9.107
LVEF	-0.679	0.048	7.345	<0.001	0.507	0.462-0.557
BUN	0.013	0.547	0.001	0.982	1.013	0.347~2.959
UA	0.267	0.258	1.075	0.300	1.306	0.788~2.164
CysC	0.503	0.209	5.809	0.016	1.654	1.099~2.491
eGFR	-0.798	0.047	10.084	<0.001	0.450	0.411-0.494
